# Burden of cancers attributable to high fasting plasma glucose in the Middle East and North Africa region, 1990–2019

**DOI:** 10.1002/cam4.5743

**Published:** 2023-03-23

**Authors:** Farhad Tondro Anamag, Maryam Noori, Seyed Aria Nejadghaderi, Mark J. M. Sullman, Jessica A. Grieger, Ali‐Asghar Kolahi, Saeid Safiri

**Affiliations:** ^1^ Social Determinants of Health Research Center, Department of Community Medicine, Faculty of Medicine Tabriz University of Medical Sciences Tabriz Iran; ^2^ Student Research Committee, School of Medicine Iran University of Medical Sciences Tehran Iran; ^3^ Urology Research Center Tehran University of Medical Sciences Tehran Iran; ^4^ Research Center for Integrative Medicine in Aging Aging Research Institute, Tabriz University of Medical Sciences Tabriz Iran; ^5^ Systematic Review and Meta‐analysis Expert Group (SRMEG) Universal Scientific Education and Research Network (USERN) Tehran Iran; ^6^ Department of Life and Health Sciences University of Nicosia Nicosia Cyprus; ^7^ Department of Social Sciences University of Nicosia Nicosia Cyprus; ^8^ Adelaide Medical School University of Adelaide Adelaide South Australia Australia; ^9^ Robinson Research Institute University of Adelaide Adelaide South Australia Australia; ^10^ Social Determinants of Health Research Center Shahid Beheshti University of Medical Sciences Tehran Iran; ^11^ Hematology and Oncology Research Center Tabriz University of Medical Sciences Tabriz Iran

**Keywords:** cancer, Eastern Mediterranean region, global burden of disease, high fasting plasma glucose, Middle East and North Africa

## Abstract

**Background:**

The present study reported the cancer deaths and disability‐adjusted life years (DALYs) that were attributable to high fasting plasma glucose (HFPG) in the Middle East and North Africa (MENA) region by country, age, sex, cancer type and Socio‐demographic Index (SDI), from 1990 to 2019.

**Methods:**

Global Burden of Disease (GBD) 2019 data were used to report the cancer‐related deaths and DALYs that were attributable to HFPG, for all MENA countries over the period 1990–2019. The results were presented as numbers, population attributable fractions (PAFs) and rates (per 100,000) with 95% uncertainty intervals.

**Results:**

In 2019, there were an estimated 19.8 thousand (5.5–40.2) cancer deaths attributable to HFPG in MENA, which represents 4.7% (1.3–9.3) of all cancer‐related deaths. The number of regional DALYs due to HFPG‐related cancers was 462.2 thousand (127.3–959.5), which represents 3.8% (1.1–7.6) of all cancer‐related DALYs in 2019. From 1990 to 2019, the age‐standardized DALY rate of cancers attributable to HFPG (per 100,000) grew from 56.3 (14.6–121.1) to 107.0 (29.8–220.8), which was a 90.1% (64.4–127.8) increase. In 2019, the highest age‐standardized DALY rate of cancers attributable to HFPG was in Qatar (270.4) and the lowest was in Sudan (75.3). The DALY rate of cancers attributable to HFPG increased with age, peaking for males in the 70–74 age group and for females in the 75–79 age group, and then decreased for both sexes. A broadly positive relationship was found between the national SDI and the national age‐standardized DALY rate of all cancers attributable to FPG over the measurement period.

**Conclusions:**

The burden of HFPG‐related cancers has increased over the past three decades in MENA, and was higher than the global average in multiple age groups. Implementing a battery of preventive measures and therapeutic interventions is suggested to address the adverse effects of this modifiable risk factor.

## Graphical abstract

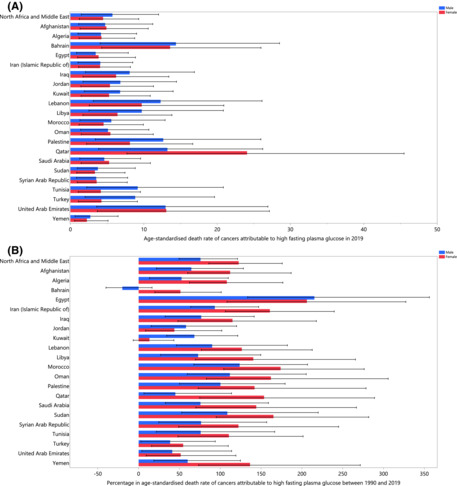

## 1 INTRODUCTION

Globally, diabetes and hyperglycaemia are common causes of disability and death, both via the direct clinical consequences and an increased risk of several non‐communicable diseases (NCDs).^1^, ^2^, ^3^ High fasting plasma glucose (HFPG) is related to a number of diseases, including neurological disorders, cardiovascular diseases, kidney impairments, respiratory infections and tuberculosis.^4^ In 2017, HFPG was globally responsible for more than 6 million deaths and 166 million disability‐adjusted life years (DALYs) from NCDs, which represents 15.6% of all deaths and 10.7% of all DALYs.^5^ Furthermore, in the Middle East and North Africa (MENA) region, HFPG was reported to be the fourth largest cause for all DALYs,^6^ accounting for 8.5% of all deaths and 4.9% of all DALYs in 2013.^7^

HFPG has been linked with multiple cancers, in terms of both the development and associated complications. These cancers include tracheal, bronchus, and lung (TBL) cancer,^8^ bladder cancer,^9^ colorectal cancer,^10^ breast cancer,^11^ liver cancer,^12^ ovarian cancer^13^ and pancreatic cancer.^14^ Previous research has evaluated the burden of cancers attributable to different risk factors using data from the global burden of disease (GBD) 2019 project. This study found that HFPG was the fifth (males) and fourth (females) largest contributors to the age‐standardized rate of cancer‐related DALYs. In addition, globally the most common cancers, in terms of HFPG‐attributable DALYs, were tracheal, bronchus and lung cancer for males and breast cancer for females.^15^ A number of previous studies have investigated the effect of HFPG and diabetes mellitus on the development, progression, management and complications associated with the different types of cancers, but there have been no previous research concerning the HFPG‐related burden of cancers in the MENA region. Previous research has examined the elevated risk that HFPG, or diabetes, has for a number of NCDs, but these have not been cancer‐specific or confined to the MENA region.^8^, ^16^ Given the heterogeneity of the countries that comprise MENA, particularly regarding the level of development, the rapidly changing nature of this region, and the scattering of high‐quality evidence, providing updated and detailed information on the burden of this disorder is vital.

The present study reported the burden of HFPG‐related cancers by age, sex, sociodemographic index (SDI), and cancer type for the 21 MENA countries between 1990 and 2019 using data from the GBD 2019 study. These findings could be helpful for resource allocation decisions, the implementation of screening programs for high‐risk patients, allowing more precise decision making in prioritizing public health measures, and informing cancer patients with diabetes about the necessity for diabetes care.

## 2 METHODS

### 2.1 Overview

The GBD project is a global epidemiological venture which investigates the burden of diseases and injuries in most countries throughout the world. The latest version of the project, GBD 2019, has collected data for 369 diseases and injuries and 87 risk factors over the period 1990 to 2019.^17^ In GBD 2019, the comparative risk assessment framework was used to model the burden of cancers attributable to HFPG. The present study aimed to report the death and DALY estimates for the 21 MENA countries by age, sex, and SDI, from 1990 to 2019. MENA is comprised of the following countries: Afghanistan, Algeria, Bahrain, Egypt, Iran (Islamic Republic of), Iraq, Jordan, Kuwait, Lebanon, Libya, Morocco, Oman, Palestine, Qatar, Saudi Arabia, Sudan, the Syrian Arab Republic, Tunisia, Turkey, the United Arab Emirates (UAE) and Yemen. A detailed explanation of the approaches used to model the HFPG‐attributable burden have previously been published in the GBD capstone articles.^4^, ^17^, ^18^ The data sources included are available online (https://ghdx.healthdata.org/gbd‐2019/data‐input‐sources) as are the results (http://ghdx.healthdata.org/gbd‐results‐tool).

### 2.2 Data sources and case definition

Fasting plasma glucose (FPG) was considered to be a continuous variable and was measured in mmol/L. The mean of FPG in the targeted population was measured to evaluate HFPG. HFPG was defined as any amount over the theoretical minimum‐risk exposure level (TMREL) of 4.8–5.4 mmol/L or 86.4–97.2 mg/dL. TMREL is defined as the level of risk exposure that keeps the risk of disease, at the population level, to a minimum.

In GBD 2019, a systematic review was performed up to 17 October 2018, which included both FPG and diabetes. The review included all research which reported the FPG and/or prevalence of diabetes. The detailed search strategy can be found in Appendix S1. The data entered into the final model were obtained from three sources: (1) mean FPG estimates in a sample population; (2) diabetes prevalence estimates in a sample population; and (3) survey data on FPG at the individual‐level. It should be noted that articles were excluded if they did not report the average FPG or the prevalence of diabetes, and the mean FPG was used when both were reported. Furthermore, individual‐level data was used, where possible, and were combined to provide estimations for each sex, age group, year and geographical location. Data sources used in the modelling/estimation process can be obtained using the GBD Data Input Sources Tool.^17^

### 2.3 Data processing and modelling

There were several stages in the data processing, in order to minimize the sampling and measurement errors and to make the data more comparable. Age‐ and sex‐ group samples that contained less than 30 individuals were deemed too small and were combined with their adjacent age group to avoid the loss of data and reduce potential bias. Studies were excluded if the total sample size was less than 30 people and no population‐weights were included. Moreover, a GBD developed probability ensemble was used to model the prevalence of diabetes using the mean FPG. Individual‐level data was utilized to describe the distribution of FPG. The reference case definition was FPG >126 mg/dL (i.e., 7 mmol/L) or the use of medication to treat diabetes.^17^, ^19^

Exposure estimates were calculated from 25 years old onwards, for both sexes, every 5‐year age group, each national and subnational location, for every year between 1990 and 2019. As was the case in earlier iterations of the GBD project, a Spatio‐Temporal Gaussian Process Regression (ST‐GPR) framework was employed to predict the average FPG at the age, sex, location and year levels.^17^ In surveys aiming to determine the prevalence of diabetes mellitus, FPG was routinely checked and reported. A glucose test and questions regarding medications to treat diabetes may also have been included, as a part of the case definition of diabetes. However, the FPG test may not have been administered to those with a history of diabetic therapy, and so the FPG mean would not be indicative of the population's mean. In these instances, the prevalence of diabetes was calculated using an FPG > 126 mg/dL (7 mmol/L) definition, before they were crosswalked to the reference definition, before the mean FPG was estimated. To inform the estimates in data‐sparse countries, IHME systematically tested a range of covariates and selected the age specific prevalence of obesity as a covariate, based on the direction of the coefficient and the significance level.

### 2.4 Data on the estimated relative risk

The TMREL for FPG was estimated to be 4.8–5.4 mmol/L or 86.4–97.2 mg/dL (CF the level required for a diabetes diagnosis ≥7.0 mmol/L or 126 mg/dL)^17^ by averaging the person‐year weighted values of FPG linked to the smallest risk of death, calculating from pooling the analyses of those studies that used a prospective cohort methodology. While substantial overlap exists between diabetes and HPFG, they are not exactly the same. For example, individuals diagnosed with pre‐diabetes have HFPG, but not diabetes, whereas some individuals with well managed diabetes may have a lower HFPG than those with pre‐diabetes. A dose–response meta‐analysis of prospective cohort studies was used to calculate the relative risks (RR). The morbidity and mortality attributable to type 1 and type 2 diabetes were regarded as being completely due to FPG. The systematic review showed that a number of cancers were related to HFPG, including cancers of the^17^: trachea, bronchus and lung,^20^ breast,^21^ bladder,^22^ ovaries,^23^ pancreas,^24^ liver^25^ and colon and rectum.^26^

### 2.5 Statistical analysis

In GBD 2019, the TMREL was used to estimate the relative risk (RR) and the risk factor levels that correlated with a reduced level of risk. The burden of HFPG‐related cancers by sex, age, location and year, were then estimated using population‐attributable fractions (PAFs). The PAF is defined as the proportion of risk that would be decreased in a particular year if the exposure to an individual risk factor were reduced to an ideal level, and for a continuous risk factor like HFPG it is expressed as:

$${\text{34𝑃34𝑃𝐴34𝑃𝐴𝐹}}_{\text{34𝑃𝐴𝐹𝑗34𝑃𝐴𝐹𝑗𝑜34𝑃𝐴𝐹𝑗𝑜𝑎34𝑃𝐴𝐹𝑗𝑜𝑎𝑠34𝑃𝐴𝐹𝑗𝑜𝑎𝑠𝑔t}}=\frac{\int_{x=l}^{u}\text{RRjoasg}\left(x\right)\text{Pjasgt}\left(x\right)\mathrm{dx}–\text{RRjoasg}\left(\text{TMRELjas}\right)}{\int_{x=l}^{u}\text{RRjoas}\left(x\right)\text{Pjasgt}\left(x\right)\mathrm{dx}\;}$$

where 𝑃𝐴𝐹𝑗𝑜𝑎𝑠𝑔𝑡 is the PAF for cause 𝑜 due to risk factor 𝑗 for age group 𝑎, sex 𝑠, location 𝑔 and year 𝑡. 𝑅𝑅𝑗𝑜𝑎𝑠𝑔(𝑥) is the RR as a function of exposure level 𝑥 for risk factor 𝑗 for cause 𝑜, age group 𝑎, sex 𝑠 and location 𝑔, with the lowest level of observed exposure as 𝑙 and the highest as 𝑢; 𝑃𝑗𝑎𝑠𝑔𝑡(𝑥) is the distribution of exposure at 𝑥 for age group 𝑎, sex 𝑠, location 𝑔 and year 𝑡; and 𝑇𝑀𝑅𝐸𝐿𝑗𝑎𝑠 is the TMREL for risk factor j, age group a and sex *s*.

In order to estimate the number of HFPG‐related deaths and the number of DALYs, the PAFs were individually multiplied with the overall number of deaths and the total number of DALYs, obtained from the GBD 2019 study, for each MENA country, sex, age group, year and cancer type. The methodology for estimating the number of deaths and DALYs attributed to cancer has previously been reported in detail^4^ and the results are provided as numbers, PAFs and age‐standardized rates/100,000 population, and these estimates all had 95% uncertainty intervals (UIs).

In addition, this research examined the relationship between HFPG‐attributable cancers and the level of socio‐economic development (SDI). SDI is a summary measure of the following three covariates: mean educational achievement for those in the country aged 15 and older, the lag‐distributed income per capita and the fertility rate among women less than 25 years of age. All components were equally weighted and SDI was calculated as the geometric mean of all of the above variables, which is scored from 0 (least developed) to 1 (most developed). SDI was initially produced for GBD 2015, based upon the Human Development Index, and has been refined for each new GBD cycle in response to feedback from GBD collaborators.^4^, ^17^

## 3 RESULTS

### 3.1 The Middle East and North Africa region

In 2019, an estimated 19.8 thousand (95% UI: 5.5 to 40.2) cancer deaths were attributable to HFPG in the region, which is 4.7% (1.3 to 9.3) of all cancer‐related deaths (Table 1). The age‐standardized death rate for HFPG‐related cancers in 2019 (5.1 [1.4–10.3] per 100,000) was 93.9% (68.3–133.3) higher than in 1990 (2.6 [0.7–5.6]) (Table S1). In 2019, there were 462.2 thousand (127.3–959.5) DALYs in MENA, representing 3.8% (1.1–7.6) of all cancer‐related DALYs. The age‐standardized DALY rate/100,000 of HFPG‐related cancers rose from 56.3 (14.6–121.1) in 1990 to 107.0 (29.8–220.8) in 2019, a relative increase of 90.1% (64.4–127.8) (Table S2).

**TABLE 1:** Cancer deaths and DALYs attributable to high fasting plasma glucose in the Middle East and North Africa (MENA) region in 2019 and the percentage change in the age‐standardized rates during the period 1990–2019 (Generated from data available from http://ghdx.healthdata.org/gbd‐results‐tool).

	Deaths (95% UI)				DALY (95% UI)			
	Counts (2019)	PAF (2019)	ASRs (2019)	% change in ASRs 1990–2019	Counts (2019)	PAF (2019)	ASRs (2019)	% change in ASRs 1990–2019
North Africa and Middle East	19,755 (5517, 40,246)	4.7 (1.3, 9.3)	5.1 (1.4, 10.3)	93.9 (68.3, 133.3)	462,151 (127,349, 959,468)	3.8 (1.1, 7.6)	107 (29.8, 220.8)	90.1 (64.4, 127.8)
Afghanistan	510 (140, 1146)	2.4 (0.7, 5)	4.8 (1.4, 10.4)	82.2 (39.9, 137.8)	13,645 (3606, 31,320)	1.7 (0.5, 3.6)	106.7 (29, 238.8)	82 (36.6, 143.6)
Algeria	1236 (353, 2587)	5.2 (1.5, 10.3)	4.2 (1.2, 8.7)	74.8 (36.2, 134.3)	27,598 (7625, 58,330)	4.1 (1.2, 8.2)	82.8 (23.3, 173.5)	78 (35.6, 143.8)
Bahrain	84 (25, 166)	10.3 (3.3, 18.9)	13.7 (4.3, 26.5)	3.2 (−19.8, 42.5)	2023 (601, 4073)	8.2 (2.6, 15.3)	240.8 (74.5, 472.1)	−3.1 (−26.2, 34.7)
Egypt	2116 (568, 4855)	3.7 (1, 7.5)	3.6 (1, 8.1)	204.4 (122.5, 323.9)	54,923 (14,509, 127,141)	3 (0.8, 6.2)	81.8 (21.8, 188.4)	209.3 (125.9, 331.7)
Iran (Islamic Republic of)	2689 (777, 5382)	4 (1.2, 8)	4.1 (1.2, 8.1)	122.7 (92.7, 174)	58,946 (16,763, 119,374)	3.3 (0.9, 6.7)	82 (23.5, 165)	121.3 (93.3, 169.7)
Iraq	1434 (410, 3032)	5.9 (1.7, 11.6)	7 (2, 14.5)	91.4 (45, 155)	35,527 (9893, 77,129)	4.6 (1.3, 9.1)	152.7 (43.7, 324.4)	84.2 (36.9, 148.5)
Jordan	335 (93, 696)	5.9 (1.7, 11.6)	6.1 (1.7, 12.6)	52.6 (23.9, 95.8)	7896 (2164, 16,738)	4.6 (1.3, 9.2)	123.4 (34.2, 256.5)	44.8 (17.1, 86.7)
Kuwait	123 (36, 248)	7.4 (2.2, 14.4)	6.2 (1.9, 12.4)	45.8 (23, 82.4)	2746 (799, 5615)	5.8 (1.7, 11.3)	116.7 (34.4, 234.9)	35.7 (14.6, 67.3)
Lebanon	564 (160, 1168)	7.5 (2.2, 14.7)	10.9 (3.1, 22.4)	104 (65.1, 189.3)	11,536 (3217, 23,874)	6.3 (1.8, 12.4)	222.1 (62, 459.5)	104.9 (61.9, 187.9)
Libya	370 (102, 777)	6.8 (2, 13.5)	8.1 (2.3, 16.8)	93.5 (48.4, 172.1)	8838 (2407, 18,898)	5.5 (1.6, 11)	175 (48, 369.2)	98.5 (50.3, 184.8)
Morocco	1504 (398, 3276)	5.4 (1.5, 11.1)	5 (1.3, 10.9)	143.5 (87.7, 221.9)	38,079 (9788, 83,922)	4.7 (1.3, 9.8)	115.5 (30.4, 251.7)	142.9 (83.3, 224.5)
Oman	62 (18, 127)	4.5 (1.3, 9)	5.2 (1.5, 10.4)	136.4 (82.7, 226.9)	1500 (420, 3094)	3.3 (1, 6.7)	99.3 (29, 201.5)	115 (67.5, 200)
Palestine	201 (57, 407)	6.9 (2, 13.5)	10 (2.9, 20)	113.6 (64.6, 198.5)	4688 (1315, 9671)	5.3 (1.5, 10.5)	205 (58, 415.5)	109.7 (59.7, 197)
Qatar	73 (22, 148)	8.8 (2.8, 16.4)	15.8 (5.1, 30.3)	74.7 (32.4, 147.6)	1974 (583, 4071)	6.9 (2.2, 13.1)	270.4 (84.1, 525.9)	57.4 (18.9, 120.9)
Saudi Arabia	680 (191, 1416)	5.2 (1.5, 10.2)	4.9 (1.4, 10)	103.6 (56.5, 184.5)	19,011 (5249, 40,435)	4.1 (1.2, 8.3)	102.4 (29.3, 211.7)	108.7 (55.6, 191.5)
Sudan	586 (165, 1318)	3.4 (1, 7.1)	3.6 (1, 7.9)	132.3 (80.4, 223.4)	13,893 (3754, 31,710)	2.5 (0.7, 5.1)	75.3 (20.9, 170.3)	132 (75.2, 225.5)
Syrian Arab Republic	381 (101, 828)	4.3 (1.2, 8.6)	3.5 (1, 7.4)	92.9 (40.2, 172)	9011 (2326, 19,813)	3.5 (1, 7)	71.8 (18.9, 156.8)	86.6 (32.9, 166)
Tunisia	785 (206, 1769)	7.8 (2.1, 15.4)	6.5 (1.7, 14.7)	80.2 (29.4, 161.7)	17,163 (4322, 39,392)	6.6 (1.8, 13.2)	134.6 (34.3, 310)	86.5 (30.8, 175.1)
Turkey	5434 (1367, 11,696)	5.2 (1.4, 10.6)	6.4 (1.6, 13.7)	43.1 (9.7, 91.7)	117,239 (29,332, 256,769)	4.4 (1.1, 9.2)	132.3 (33.2, 289.2)	33.9 (1.2, 80.4)
United Arab Emirates	280 (77, 588)	5.2 (1.5, 10.4)	12.8 (3.8, 25.7)	46.3 (13.2, 99.4)	8432 (2275, 18,166)	4 (1.2, 8.2)	247.8 (72.4, 501)	48.8 (13.1, 105.7)
Yemen	286 (74, 644)	2.3 (0.6, 4.8)	2.5 (0.7, 5.4)	97.7 (55.9, 169.9)	7011 (1784, 16,125)	1.7 (0.4, 3.6)	52.8 (13.4, 119.9)	96.2 (48.7, 171.3)

Abbreviations: ASRs, Age‐standardized rates; DALY, Disability‐adjusted life year; GBD, Global Burden of Disease; PAF, Population attributable fraction; UI, Uncertainty interval.

### 3.2 National level

**FIGURE 1:**
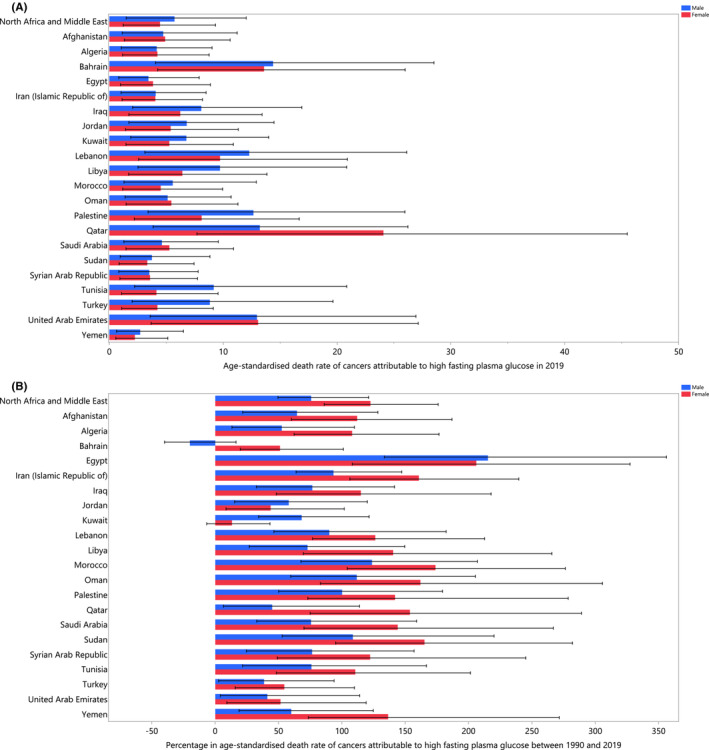
Age‐standardized death rate (per 100,000) in 2019 (A) and the percentage change in the age‐standardized death rate from 1990 to 2019 (B) rates of cancer deaths attributable to high fasting plasma glucose in the Middle East and North Africa (MENA) region, by sex and country.

The PAFs of all deaths from HFPG‐related cancers ranged from 2.3% to 10.3%. Bahrain (10.3% [3.3–18.9]), Qatar (8.8% [2.8–16.4]) and Tunisia (7.8% [2.1–15.4]) had the three largest PAFs, while the smallest were in Yemen (2.3% [0.6–4.8]), Afghanistan (2.4% [0.7–5.0]) and Sudan (3.4% [1.0–7.1]) (Table 1). The age‐standardized deaths (per 100,000) in 2019 were highest in Qatar (15.8 [5.1–30.3]), Bahrain (13.7 [4.3–26.5]) and the UAE (12.8 [3.8–25.7]). The lowest rates were found in Yemen (2.5 [0.7–5.4]), the Syrian Arab Republic (3.5 [1.0–7.4]) and Sudan (3.6 [1.0–7.9]) (Table 1). Figure 1A presents the 2019 age‐standardized death rates for the different types of cancers that were attributable to HFPG, by sex. The age‐standardized death rates increased in almost all MENA countries, from 1990 to 2019, with the largest increases occurring in Egypt (204.4% [122.5–323.9]), Morocco (143.5% [87.7–221.9]) and Oman (136.4% [82.7–226.9]) (Table 1). The changes in the age‐standardized death rate over the measurement period are presented, by sex, in Figure 1B.

In 2019, the percentage of all HFPG‐related cancer DALYs ranged from 1.7% to 8.2%. Bahrain (8.2% [2.6–15.3]), Qatar (6.9% [2.2–13.1]) and Tunisia (6.6% [1.8–13.2]) had the three largest PAFs, while the smallest were found in Yemen (1.7% [0.4–3.6]), Afghanistan (1.7% [0.5–3.6]) and Sudan (2.5% [0.7–5.1]) (Table 1). Figure S1 presents the 2019 age‐standardized DALY rates by sex. In 2019, the age‐standardized DALY rates were highest in Qatar (270.4 [84.1–525.9]), the UAE (247.8 [72.4–501.0]) and Bahrain (240.8 [74.5–472.1]). Conversely, the lowest rates were found in Yemen (52.8 [13.4–119.9]), the Syrian Arab Republic (71.8 [18.9–156.8]) and Sudan (75.3 [20.9–170.3]) (Table 1). The age‐standardized DALY rates increased for most countries between 1990 and 2019, with the largest being found in Egypt (209.3% [125.9–331.7]), Morocco (142.9% [83.3–224.5]) and Sudan (132.0% [75.2–225.5]) (Table 1). Figure S2 presents the changes in the age‐standardized DALY rate from 1990 to 2019 by sex.

**FIGURE 2:**
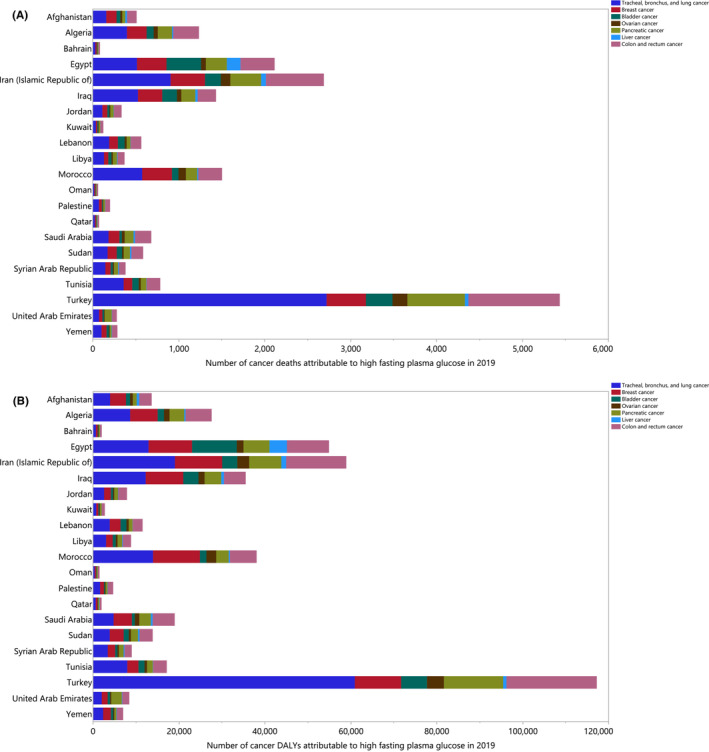
Number of cancer deaths (A) and DALYs (B) related to high fasting plasma glucose in 2019 by cancer type in the Middle East and North Africa (MENA) region. DALY, disability‐adjusted life years.

In 2019, there were large differences in the death and DALY counts that were attributable to HFPG‐related cancers. The largest number of deaths and DALYs, from HFPG‐related cancers, were due to tracheal, bronchus and lung (TBL) cancers. Turkey, Iran and Morocco had the most deaths and DALYs due to TBL cancer. In most MENA countries, colon and rectum cancer accounted for the second highest number of deaths and DALYs, and breast cancer was third in almost all MENA countries (Figure 2A,B).

### 3.3 Sex and age patterns

**FIGURE 3:**
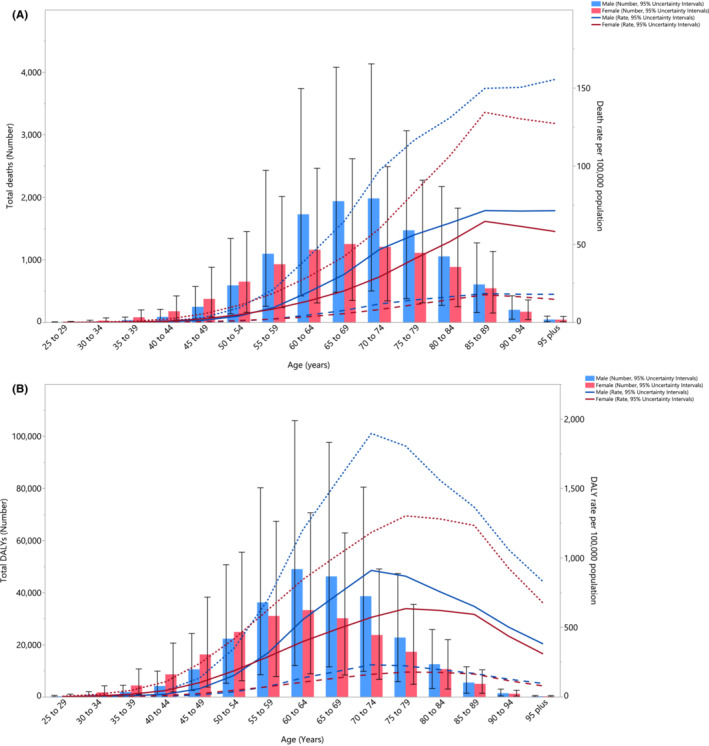
Number of deaths and death rate (A) and the global number of DALYs and DALY rate (B) of cancers attributable to high fasting plasma glucose (per 100,000) by age and sex in the Middle East and North Africa (MENA) region in 2019; Dotted lines show the 95% upper uncertainty interval and the dashed lines the lower. DALY, disability‐adjusted life years.

In 2019, the largest number of HFPG‐related cancer deaths were found among 70–74 year olds, for both men and women. The death rate from HFPG‐related cancers increased constantly, for both males and females, until the 85–89 year old age group (for both sexes) and then started to gently decrease. There were no substantial sex differences, regarding the number of deaths and the death rate (Figure 3A). Moreover, the regional number of DALYs in 2019 were highest among 60–64 year olds for both sexes. The DALY rate began rising in the young age groups, before peaking among the 70–74 year olds for males and the 75–79 year olds for females, and then decreased for both sexes (Figure 3B).

**FIGURE 4:**
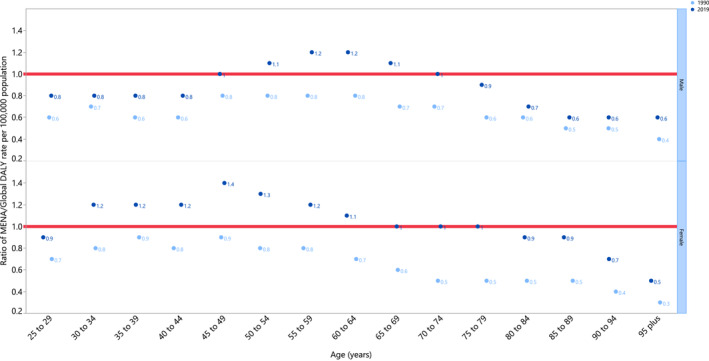
Ratio of the Middle East and North Africa (MENA) region to the global age‐standardized DALY rate of cancers attributable to high fasting plasma glucose (per 100,000) by age group and sex, 1990–2019. DALY, disability‐adjusted life years.

In 2019, the MENA /global DALY rate (per 100,000) ratio varied according to sex and age group. The MENA region's age‐standardized DALY rates were higher than the corresponding global rate for the 50–69 and 30–64 age groups for males and females respectively. In 2019, the DALY rate was equal to the global rate for 70–74 year old males and 65–79 year old females. In 1990, the MENA age‐standardized DALY rate was consistently lower than the global rate, for both males and females (Figure 4).

### 3.4 Association with the socio‐demographic Index (SDI)

**FIGURE 5:**
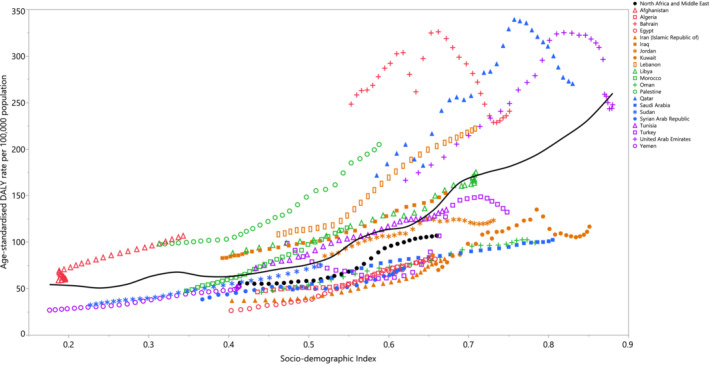
Age‐standardized DALY rates of cancers attributable to high fasting plasma glucose for the 21 Middle East and North Africa (MENA) countries by Socio‐demographic Index, 2019; the black line shows the expected values, based upon the Socio‐demographic Index and disease rates. Thirty points are plotted for each MENA country and show the observed age‐standardized DALY rates from 1990 to 2019. Data points above the solid black line showed a higher than expected burden, while those below the line showed a lower than expected burden (both based on SDI). DALY, disability‐adjusted life years.

SDI was positively related to the age‐standardized DALY rates for all HFPG‐related cancers over the measurement period (1990–2019). Data points above the solid black line indicate a burden that was higher than expected, while the data points below the line indicate a burden that was lower than expected (both based on SDI). Qatar, Bahrain, Lebanon, Palestine, Iraq and Afghanistan had higher than expected burdens, while Yemen, Egypt, Algeria, Kuwait, Sudan, Iran, the Syrian Arab Republic, Oman and Saudi Arabia were lower than expected across the entire measurement period. Moreover, during the measurement period, Turkey, Jordan, Tunisia, the UAE and Libya changed from a higher than expected to a lower than expected rate, while Morocco reached a higher than expected rate (Figure 5).

## 4 DISCUSSION

The current research reported the number of deaths, DALYs and the age‐standardized rates that were attributable to HFPG‐related cancers in the MENA region from 1990 to 2019. In 2019, there were approximately 20,000 deaths and 462,000 DALYs in the MENA region, accounting for 4.7 and 3.8% of all HFPG‐related deaths and DALYs from cancer respectively. Our findings showed there was an almost 90% increase in the HFPG‐related burden of cancers in this region between 1990 and 2019. Furthermore, there was a positive correlation between the age‐standardized DALY rate and the SDI.

The present research is the first to describe the burden of HFPG‐related cancers in the MENA region. Since there are few studies reporting the HFPG‐related burden of cancers at the regional and global levels, it is difficult to make an accurate comparison with previous findings.^16^ However, the GBD 2017 reported that the global burden of HFPG‐related non‐communicable diseases decreased with time, was higher in males, and was negatively associated with SDI.^8^ In contrast, we found an increase in the HFPG‐related burden of cancers with time, no substantial sex differences and a positive association with developmental level (i.e. SDI). When we compared the HFPG‐attributable burden of cancers in MENA with the global estimates, we found that from 1990 to 2019 the DALY rates exceeded the global average. The higher burden could be because of the greater prevalence of diabetes and obesity in MENA, along with the lack of effective programs for managing cancer patients with HFPG, which complicates the course of the disease and causes ongoing disabilities and deaths.^27^, ^28^

Furthermore, our results showed an increase of more than 90% in the age‐standardized cancer deaths and DALY rates in MENA, which is broadly in agreement with a reported 85.5% increase in the prevalence of type 2 diabetes in this region over the last 30 years.^27^ Moreover, we found that Bahrain and Qatar had considerably higher PAFs than those found in the rest of the MENA region, which is in line with their higher prevalence of diabetes.^27^ Likewise, Egypt and Morocco had the largest increases, from 1990 to 2019, in the prevalence of type 2 diabetes, which is consistent with our findings in the age‐standardized death and DALY rates.^27^ These observations highlight the importance of incorporating effective public health interventions and preventive measures in these countries. On the other hand, the low rate of cancer deaths and DALYs attributed to HFPG in Yemen and Syria could be explained in part by the poor health infrastructures that are as a result of civil wars, the emigration of experienced health professionals and health system corruption in these countries, which have led to low disease detection rates.^29^, ^30^, ^31^

Another reason for the large inter‐country differences in the burden of HFPG‐related cancers in the MENA region could be due to differences in the inter‐country levels of overweight and obesity. It has been established that having a high body mass index raises the likelihood of insulin resistance and subsequently results in elevated blood glucose.^32^ Globally, the prevalence of overweight and obesity among adults increased by 27.5% between 1980 and 2013. During the same period, increases of 24.8% in adult males and 25.7% in adult females were reported in the MENA region.^33^ Qatar, which had the highest burden of HFPG‐related cancers, was among the top three MENA countries for excess body weight. In contrast, Yemen had the lowest burden of HFPG‐related cancers and diabetes, and also had the lowest obesity and overweight prevalence in the MENA region.^33^

A major factor influencing cancer DALYs and deaths in those with comorbid diabetes is that these patients often have complications with infections that are characterized by shorter remission periods and an overall poorer prognosis.^34^ This could be attributed, in part, to the side effects of chemotherapeutic agents, which exacerbate the microvascular and macrovascular complications of diabetes, such as neurotoxicity (e.g., cisplatin), nephrotoxicity (e.g. cyclophosphamide) and cardiotoxicity (e.g., doxorubicin).^35^, ^36^, ^37^ Furthermore, as chemotherapy progresses diabetic patients show less compliance with the diabetes self‐management program.^38^ In addition, given the use of glucocorticoids in multiple chemotherapy protocols, managing patients' blood glucose levels becomes more challenging.^39^ For these reasons, diabetic patients with cancer should be admitted to tertiary hospitals with specialized physicians for the proper management of this urgent condition.

The GBD study provided estimates of the HFPG‐related cancer deaths and DALYs for a number of tumour types. Several studies have focused on the relationship type 2 diabetes has with the risk of developing cancer, at a number of different sites, as well as their prognosis.^39^, ^40^ In an umbrella review, Tsilidis and colleagues revealed that only a few of the reported associations between diabetes and cancers had reliable causal links without evidence of bias. The only relationships which had strong evidence, regarding the development of cancer following a diagnosis of diabetes, included breast, intrahepatic cholangiocarcinoma, colorectal, endometrial and gallbladder cancer.^41^ According to a review of 97 prospective studies, diabetes decreased the survival rate of patients with the following cancer types: breast, bladder, liver, ovary, lung, colorectal and pancreas, which aligns with our findings.^42^ After taking into account the many risk factors shared by diabetes and cancer, including overweight or obesity, smoking and low physical activity, research indicates that diabetes may be an independent risk factor for cancer development and mortality.^40^, ^43^, ^44^

While some epidemiological evidence has found HFPG to be strongly related to several types of cancers, the biological mechanisms underlying these relationships are not currently known.^45^, ^46^ A number of pathways have been proposed regarding the effect of HFPG on the incidence and progression of cancer, including via altering cell proliferation and apoptosis through impairing the regulation of inflammatory cytokines, sex hormones, insulin/insulin growth factor axis and no metabolism.^46^, ^47^, ^48^, ^49^, ^50^, ^51^ Given the substantial burden of cancers,^52^, ^53^ this causal association could be of great importance for policy making in public health, and measures should be taken to alleviate the cancer attributable burden imposed on health systems.^54^, ^55^

Our results showed that the number of HFPG‐related TBL cancer deaths was high in most of the MENA countries, which is in line with the overall high number of TBL cancer deaths.^56^ These results differ from cancer rankings by total death in some of the MENA countries, like Iran, Iraq, Saudi Arabia and Oman.^57^, ^58^ This discrepancy could be partly explained by the different PAFs associated with the potential risk factors, most notably HFPG for the various cancer types.

Low physical activity and poor diet are the two most important risk factors that should first be targeted for the primary prevention of HFPG and subsequently diabetes. From 1990 to 2015, the overall ranking of DALYs caused by diets low in whole grains and little physical activity increased in the MENA region.^6^ As a result, urgent measures are needed for reducing these risk factors. Establishing higher levels of walkability and developing green spaces in the living environments could be multi‐sector policies that lower the burden caused by diabetes and HFPG.^59^ As the rural/urban ratio is diverse in the MENA countries, with Yemen being more rural and Qatar being mostly urban, this should also be taken into account. Countries with a higher socioeconomic level have more access to western nutritional patterns, which consists of energy‐dense and processed foods. The western diet has a higher risk of diabetes, metabolic syndrome and obesity.^60^, ^61^ Public health efforts should focus on tailored nutrition education regarding the consumption of healthier foods and increasing the daily level of physical activity.

In addition, it is necessary to consider the cultural background of the countries in MENA, when developing interventions to manage diabetes and HFPG. Several countries have a strong tendency to use complementary medicine and herbs, as shown by the fact that 32.18% of people in Saudi Arabia with diabetes use herbal medicines.^62^ However, since there is very little evidence to support the efficacy of most herbal therapies, the reliance on herbal medicines could be an obstacle to managing diabetes and HFPG.

As individuals with HFPG and diabetes are more likely to suffer from several types of cancers, screening programs should be undertaken more frequently. Furthermore, several studies have reported that patients suffering from diabetes receive fewer cancer screening services than their non‐diabetic counterparts. This discrepancy could be due to the time limits for medical care appointments, a perceived decline in life expectancy and cultural barriers to health education.^35^, ^63^, ^64^, ^65^, ^66^ These gaps should be taken into consideration by clinicians, in order to evaluate their patients' primary health care and to encourage them to undergo regular screening. As the burden of HFPG‐related cancers increases in the MENA region, the more important it is to implement effective preventative measures, detect cancers early and provide timely management. Interventions to reduce the prevalence of diabetes, via modifying its potential underlying factors, could be helpful in controlling the burden of HFPG‐related cancers.

### 4.1 Limitations

The current research is the only study to report the HFPG‐attributable burden of cancers, and the associated trends over a 30‐year period. However, as with all GBD studies, one large problem is the scarcity of comprehensive high quality data, which necessitates the use of modelling strategies to estimate the results. The modelling process can over or underestimate the disease burden. Furthermore, since MENA is comprised of heterogeneous counties with significant inter‐country variability, controlling for the different covariates was challenging. Moreover, other cancer contributing factors like genetic and lifestyle factors (e.g., smoking/occupational hazards) and the effect of the type of diabetes (i.e., type 1 or type 2 diabetes mellitus) could not be evaluated here, and thus additional research is needed to investigate the impact of these risk factors. Therefore, it is recommended that our results are interpreted with some degree of caution. Also, as HFPG is often asymptomatic and most of the studies have been conducted among the diabetic population, most cases had high blood glucose levels, raising the probability of underestimating the number/ proportion of prediabetes cases. Regarding the limitation, it should be noted that the PAF calculation is primarily affected by the RR and the distribution of exposure. The GBD study usually assumed that the computed RRs applied equally to both elements of mortality or morbidity for risk‐outcome pairs when there was evidence for just one component. The separate RRs were also used for each mortality and morbidity since there was evidence of statistically different RRs for death and morbidity. It was not discovered that mortality RRs were consistently greater or lower than morbidity RRs.^4^ We also suggest that future GBD iterations take these limitations into consideration.

## 5 CONCLUSION

The burden of HFPG‐related cancers has increased in the MENA countries over the last three decades, and was higher than the global average in multiple age groups. The present findings suggest implementing a battery of preventive measures, such as screening programs and therapeutic interventions to address the adverse effects of this modifiable risk factor. Moreover, health professionals should be trained in managing diabetes in patients with cancer, especially in low to middle income countries. Further studies with high methodological quality are needed to allow more accurate estimates of the burden of HFPG‐related cancers at the national and sub‐national levels.

## AUTHOR CONTRIBUTIONS

**Farhad Tondro Anamag:** Conceptualization (equal); methodology (equal); visualization (equal); writing – original draft (equal); writing – review and editing (equal). **Maryam Noori:** Writing – original draft (equal); writing – review and editing (equal). **Seyed Aria Nejadghaderi:** Writing – original draft (equal); writing – review and editing (equal). **Mark Sullman:** Writing – original draft (equal); writing – review and editing (equal). **Jessica A. Grieger:** Methodology (equal); writing – original draft (equal); writing – review and editing (equal). **Ali‐Asghar Kolahi:** Conceptualization (equal); data curation (equal); project administration (equal); resources (equal); supervision (equal); validation (equal); writing – review and editing (equal). **Saeid Safiri:** Conceptualization (equal); data curation (equal); formal analysis (equal); funding acquisition (equal); investigation (equal); methodology (equal); project administration (equal); resources (equal); software (equal); supervision (equal); writing – review and editing (equal).

## FUNDING INFORMATION

The GBD study was funded by the Bill and Melinda Gates Foundation, but they had no involvement in undertaking this research or preparing the article. The Shahid Beheshti University of Medical Sciences also provided a grant (Grant number 29055).

## CONFLICT OF INTEREST STATEMENT

The authors have no conflict of interest to declare.

## DECLARATION

None of the authors listed on the manuscript are employed by a government agency that has a primary function other than research and/or education. Also, none of the authors are submitting this manuscript as an official representative or on behalf of the government.

## ETHICS APPROVAL AND CONSENT TO PARTICIPATE

The Ethics Committees of the Tabriz University of Medical Sciences (IR.TBZMED.REC.1400.990) and the Shahid Beheshti University of Medical Sciences (IR.SBMU.RETECH.REC.1400.431) approved this research.

## CONSENT FOR PUBLICATION

Not needed.

## PATIENTS AND PUBLIC INVOLVEMENT

Patients and the public were not involved it the analyses or preparation of this manuscript.

## AUTHOR NOTE

The present article, produced using publicly available data, concerns the view of its authors, which may not be shared by the Institute for Health Metrics and Evaluation.

## Supporting information


Figure S1.

Figure S2.

Table S1.

Table S2.

Appendix S1.

## Data Availability

The data used for these analyses are available at http://ghdx.healthdata.org/gbd‐results‐tool.
