# Supplementary figures and images for: Burden of cancers attributable to high fasting plasma glucose in the Middle East and North Africa region, 1990–2019
Source: Cancer Med. 2023 Mar 23;12(8):10031–44. doi: 10.1002/cam4.5743 (PMC10166946; doi:10.1002/cam4.5743)

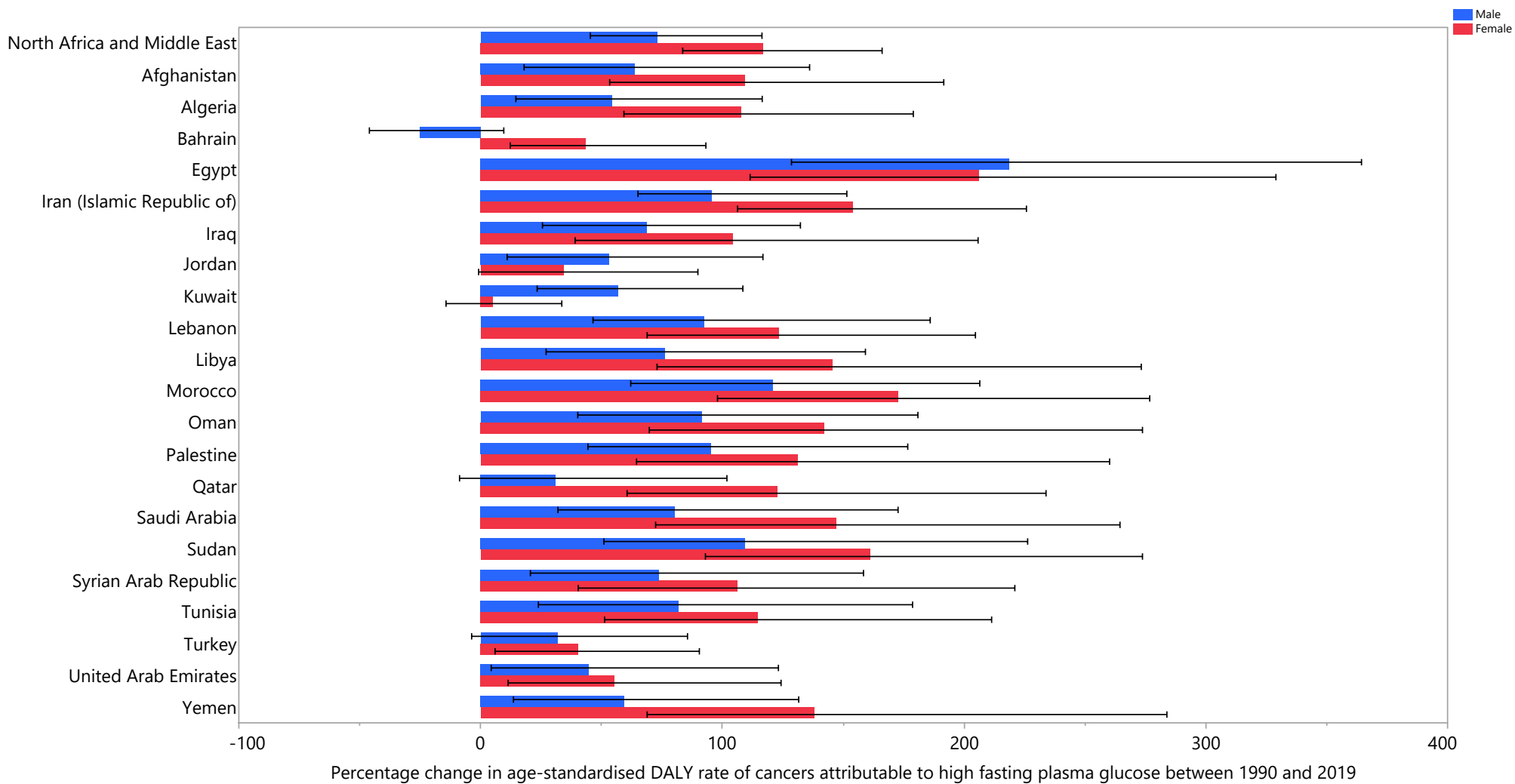

Supplement: Supplementary file 1 — Figure S1. [file CAM4-12-10031-s004.pdf]

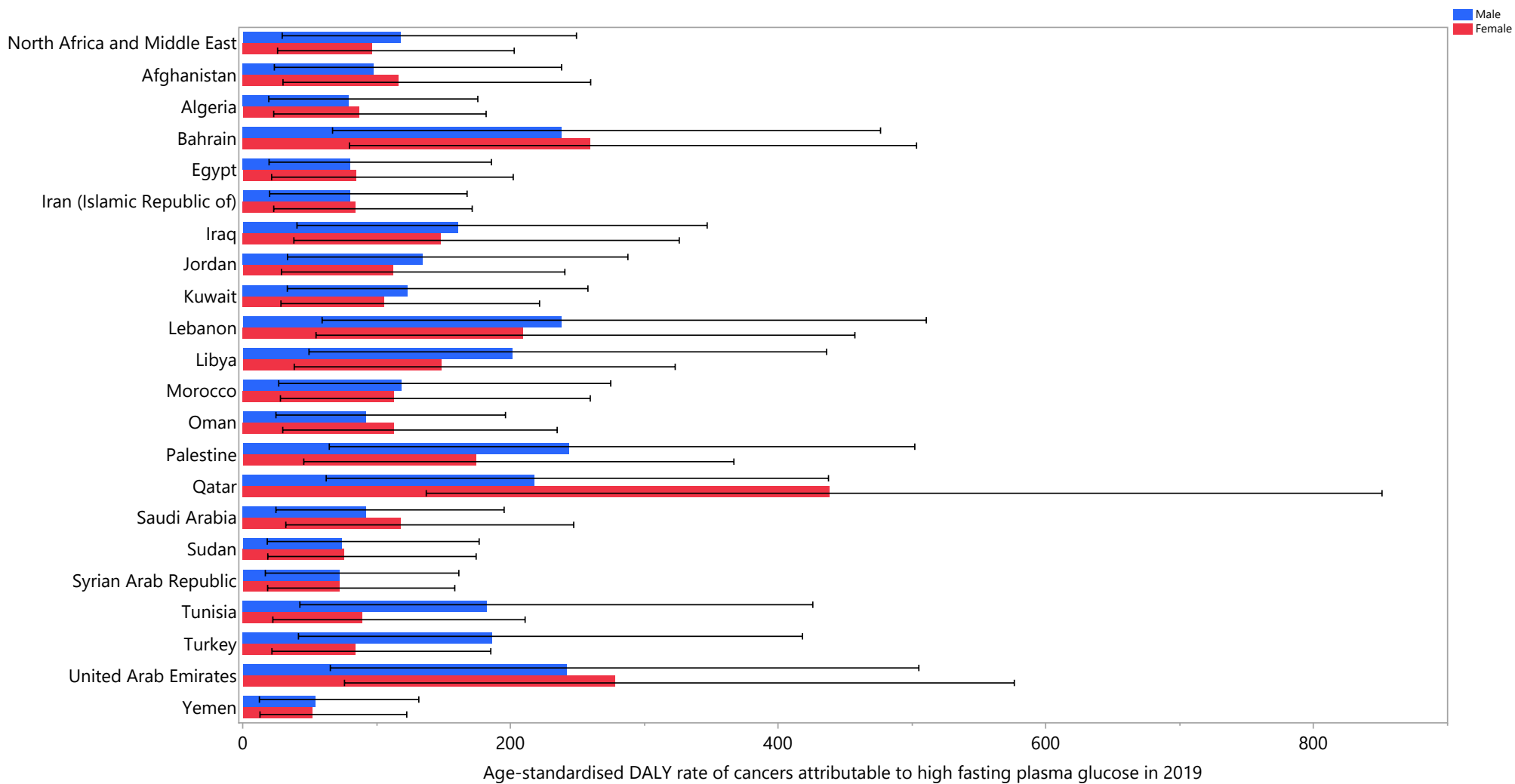

Supplement: Supplementary file 2 — Figure S2. [file CAM4-12-10031-s003.pdf]
