# Supplementary material for: Burden of cancers attributable to high fasting plasma glucose in the Middle East and North Africa region, 1990–2019
Source: Cancer Med. 2023 Mar 23;12(8):10031–44. doi: 10.1002/cam4.5743 (PMC10166946; doi:10.1002/cam4.5743)
Supplement: Supplementary file 3 — Table S1. [file CAM4-12-10031-s001.doc]

| **Table S1: Number, proportion and age-standardised rates of cancer deaths attributable to high fasting plasma glucose (per 100,000) in the Middle East and North Africa (MENA) region in 1990 and 2019 (Generated from data available from http://ghdx.healthdata.org/gbd-results-tool)** | | | | | | | |
| --- | --- | --- | --- | --- | --- | --- | --- |
|  | **1990** | | | **2019** | | | **% change in ASRs per 100,000**  **1990-2019** |
|  | **No**  **(95% UI)** | **PAF**  **(95% UI)** | **ASRs per 100,000 (95% UI)** | **No**  **(95% UI)** | **PAF**  **(95% UI)** | **ASRs per 100,000 (95% UI)** |
| **North Africa and Middle East** | **4023 (1032 , 8604)** | **2.2 (0.6 , 4.7)** | **2.6 (0.7 , 5.6)** | **19755 (5517 , 40246)** | **4.7 (1.3 , 9.3)** | **5.1 (1.4 , 10.3)** | **93.9 (68.3 , 133.3)** |
| **Afghanistan** | **175 (42 , 410)** | **1.5 (0.4 , 3.3)** | **2.6 (0.7 , 6.1)** | **510 (140 , 1146)** | **2.4 (0.7 , 5)** | **4.8 (1.4 , 10.4)** | **82.2 (39.9 , 137.8)** |
| **Algeria** | **239 (62 , 526)** | **2.3 (0.6 , 4.7)** | **2.4 (0.6 , 5.2)** | **1236 (353 , 2587)** | **5.2 (1.5 , 10.3)** | **4.2 (1.2 , 8.7)** | **74.8 (36.2 , 134.3)** |
| **Bahrain** | **17 (5 , 34)** | **6.5 (1.8 , 12.8)** | **13.3 (3.7 , 26.5)** | **84 (25 , 166)** | **10.3 (3.3 , 18.9)** | **13.7 (4.3 , 26.5)** | **3.2 (-19.8 , 42.5)** |
| **Egypt** | **312 (84 , 651)** | **1.3 (0.4 , 2.8)** | **1.2 (0.3 , 2.4)** | **2116 (568 , 4855)** | **3.7 (1 , 7.5)** | **3.6 (1 , 8.1)** | **204.4 (122.5 , 323.9)** |
| **Iran (Islamic Republic of)** | **389 (100 , 844)** | **1.5 (0.4 , 3.1)** | **1.8 (0.5 , 3.9)** | **2689 (777 , 5382)** | **4 (1.2 , 8)** | **4.1 (1.2 , 8.1)** | **122.7 (92.7 , 174)** |
| **Iraq** | **267 (69 , 590)** | **3.4 (0.9 , 7.1)** | **3.7 (1 , 8.1)** | **1434 (410 , 3032)** | **5.9 (1.7 , 11.6)** | **7 (2 , 14.5)** | **91.4 (45 , 155)** |
| **Jordan** | **45 (12 , 95)** | **3.3 (0.9 , 6.6)** | **4 (1.1 , 8.3)** | **335 (93 , 696)** | **5.9 (1.7 , 11.6)** | **6.1 (1.7 , 12.6)** | **52.6 (23.9 , 95.8)** |
| **Kuwait** | **20 (6 , 40)** | **4 (1.1 , 8)** | **4.3 (1.2 , 8.4)** | **123 (36 , 248)** | **7.4 (2.2 , 14.4)** | **6.2 (1.9 , 12.4)** | **45.8 (23 , 82.4)** |
| **Lebanon** | **108 (28 , 232)** | **3.7 (1 , 7.6)** | **5.3 (1.4 , 11.3)** | **564 (160 , 1168)** | **7.5 (2.2 , 14.7)** | **10.9 (3.1 , 22.4)** | **104 (65.1 , 189.3)** |
| **Libya** | **70 (18 , 156)** | **3.5 (0.9 , 7.3)** | **4.2 (1.1 , 9.3)** | **370 (102 , 777)** | **6.8 (2 , 13.5)** | **8.1 (2.3 , 16.8)** | **93.5 (48.4 , 172.1)** |
| **Morocco** | **266 (66 , 583)** | **2.4 (0.6 , 5.1)** | **2.1 (0.5 , 4.5)** | **1504 (398 , 3276)** | **5.4 (1.5 , 11.1)** | **5 (1.3 , 10.9)** | **143.5 (87.7 , 221.9)** |
| **Oman** | **12 (3 , 27)** | **2.1 (0.5 , 4.4)** | **2.2 (0.6 , 4.9)** | **62 (18 , 127)** | **4.5 (1.3 , 9)** | **5.2 (1.5 , 10.4)** | **136.4 (82.7 , 226.9)** |
| **Palestine** | **37 (9 , 83)** | **3.3 (0.9 , 7)** | **4.7 (1.2 , 10.3)** | **201 (57 , 407)** | **6.9 (2 , 13.5)** | **10 (2.9 , 20)** | **113.6 (64.6 , 198.5)** |
| **Qatar** | **6 (2 , 12)** | **4.4 (1.3 , 8.8)** | **9 (2.6 , 18.6)** | **73 (22 , 148)** | **8.8 (2.8 , 16.4)** | **15.8 (5.1 , 30.3)** | **74.7 (32.4 , 147.6)** |
| **Saudi Arabia** | **118 (31 , 264)** | **2.8 (0.8 , 5.8)** | **2.4 (0.6 , 5.3)** | **680 (191 , 1416)** | **5.2 (1.5 , 10.2)** | **4.9 (1.4 , 10)** | **103.6 (56.5 , 184.5)** |
| **Sudan** | **129 (32 , 314)** | **1.5 (0.4 , 3.4)** | **1.5 (0.4 , 3.7)** | **586 (165 , 1318)** | **3.4 (1 , 7.1)** | **3.6 (1 , 7.9)** | **132.3 (80.4 , 223.4)** |
| **Syrian Arab Republic** | **84 (22 , 188)** | **1.8 (0.5 , 3.9)** | **1.8 (0.5 , 4)** | **381 (101 , 828)** | **4.3 (1.2 , 8.6)** | **3.5 (1 , 7.4)** | **92.9 (40.2 , 172)** |
| **Tunisia** | **164 (39 , 355)** | **4 (1 , 8.5)** | **3.6 (0.9 , 7.9)** | **785 (206 , 1769)** | **7.8 (2.1 , 15.4)** | **6.5 (1.7 , 14.7)** | **80.2 (29.4 , 161.7)** |
| **Turkey** | **1488 (356 , 3300)** | **2.7 (0.7 , 5.9)** | **4.4 (1.1 , 9.8)** | **5434 (1367 , 11696)** | **5.2 (1.4 , 10.6)** | **6.4 (1.6 , 13.7)** | **43.1 (9.7 , 91.7)** |
| **United Arab Emirates** | **21 (6 , 44)** | **3.3 (0.9 , 6.6)** | **8.8 (2.4 , 18.2)** | **280 (77 , 588)** | **5.2 (1.5 , 10.4)** | **12.8 (3.8 , 25.7)** | **46.3 (13.2 , 99.4)** |
| **Yemen** | **53 (13 , 125)** | **1.2 (0.3 , 2.6)** | **1.2 (0.3 , 2.9)** | **286 (74 , 644)** | **2.3 (0.6 , 4.8)** | **2.5 (0.7 , 5.4)** | **97.7 (55.9 , 169.9)** |
| **ASRs: Age-standardised rates; UI: Uncertainty interval; PAF: Population attributable fraction** | | | | | | | |
