# Supplementary material for: Burden of cancers attributable to high fasting plasma glucose in the Middle East and North Africa region, 1990–2019
Source: Cancer Med. 2023 Mar 23;12(8):10031–44. doi: 10.1002/cam4.5743 (PMC10166946; doi:10.1002/cam4.5743)
Supplement: Supplementary file 5 — Appendix S1. [file CAM4-12-10031-s005.docx]

**Supplementary File 1.** The search terms used for conducting the systematic review.

Search terms: Diabetes Mellitus search string: (diabetes[TI] AND (prevalence[TIAB] OR incidence[TIAB])) OR ('Diabetes Mellitus'[MeSH Terms] AND 'epidemiology'[MeSH Terms]) OR (diabetes[TI] AND 'epidemiology'[MeSH Terms]) NOT gestational[All Fields] NOT ('neoplasms'[MeSH Terms] OR 'neoplasms'[All Fields] OR 'cancer'[All Fields]) NOT ('mice'[MeSH Terms] OR 'mice'[All Fields]) NOT ('schizophrenia'[MeSH Terms] OR 'schizophrenia'[All Fields]) NOT ('emigrants and immigrants'[MeSH Terms] OR ('emigrants'[All Fields] AND 'immigrants'[All Fields]) OR 'emigrants and immigrants'[All Fields] OR 'immigrants'[All Fields]) NOT ('pregnancy'[MeSH Terms] OR 'pregnancy'[All Fields] OR 'gestation'[All Fields]) NOT ('rats'[MeSH Terms] OR 'rats'[All Fields] OR 'rat'[All Fields]) NOT ('kidney'[MeSH Terms] OR 'kidney'[All Fields]) NOT renal[All Fields] NOT ('vitamins'[Pharmacological Action] OR 'vitamins'[MeSH Terms] OR 'vitamins'[All Fields] OR 'vitamin'[All Fields]) AND ((“glucose”[Mesh] OR “hyperglycemia”[Mesh] OR “prediabetic state”[Mesh]) AND "Geographic Locations"[Mesh] NOT "United States"[Mesh]) AND ("humans"[Mesh] AND "adult"[MeSH]) AND ("Data Collection"[Mesh] OR "Health Services Research"[Mesh] OR "Population Surveillance"[Mesh] OR "Vital statistics"[Mesh] OR "Population"[Mesh] OR "Epidemiology"[Mesh] OR surve*[TiAb]) NOT Comment[ptyp] NOT Case Reports[ptyp]) NOT "hospital"[TiAb]
